# The Attitude of Patients from a Romanian Tertiary Cardiology Center as Regards Participation in Biomarker-Based Clinical Trials – Survey Methodology

**DOI:** 10.25122/jml-2018-0072

**Published:** 2018

**Authors:** Miruna Mihaela Micheu, Oana-Maria Udrea, Mihaela Octavia Popa, Iulia Rusu, Oana Gheorghe-Fronea, Alexandru Scafa-Udriste, Maria Dorobantu

**Affiliations:** 1. Department of Cardiology, Clinical Emergency Hospital of Bucharest, Bucharest, Romania; 2. Department of Psychology, “Grigore Alexandrescu” Clinical Hospital of Bucharest, Bucharest, Romania; 3. University of Medicine and Pharmacy Carol Davila, Bucharest, Romania

**Keywords:** Informed consent, biomarker, clinical trials, patients’ attitude, methodology, Clinical trials (CTs), Informed consent form (ICF)

## Abstract

One of the challenges faced when conducting a clinical trial is the recruitment of the proposed number of participants. Accordingly, identifying barriers to patients’ enrollment and developing effective strategies to overcome them is mandatory. One of the main strategies employed to improve participation rate consists of designing the informed consent forms based on patients’ feedback. This survey aims to explore the attitude of patients admitted in a Romanian tertiary cardiology center to take part in biomarker-based clinical trials.

This is a descriptive, prospective and longitudinal single-center study. Participants will be recruited until the planned sample size will be reached (n=333). The patients will be interviewed based on a semi-structured questionnaire which includes four sections: demographics (7 items), personal medical history (7 items), attitudes (9 items) and trust (4 items). Descriptive statistics will be used to illustrate patients’ demographics, medical history, attitudes toward biomarker-based clinical trials and trust in medical researchers. Logistic regression models will be employed to assess relations between patients’ attitudes, trust, and different socio-demographic variables.

Data analysis will offer answers to key questions addressed by this survey: What amount of and in what form should information be disclosed? Who should make the invitation to participate?

The information gained will facilitate tailoring informed consent forms to suit the needs of patients with various demographic, social and educational backgrounds.

## Introduction

Latest advances in decoding patients’ “omic” data have opened up new opportunities for precision medicine to be applied in clinical practice. Patient-tailored management relies on validated biomarkers to prompt specific therapeutic interventions. Still, finding robust biomarkers remains one of the key challenges in clinical research. Well-designed studies including a large number of subjects within different socio-demographic categories are the cornerstone of biomarker identification and validation. An informed consent form (ICF) addressing the patients’ needs is a prerequisite for the patients’ agreement to participate in clinical trials (CTs). Other determinants of participation are the extent to which individuals understand the given information and the trust in medical doctors conducting the study [1, 2]. In this regard, extensive research has been conducted worldwide in order to improve the design of ICF based on patients’ feedback [3–7]. However, there is no data about the attitude of Romanian patients regarding participation in biomarker-based CTs. Therefore, the purpose of this observational study is to explore the attitude of patients admitted in a Romanian tertiary cardiology center to take part in biomarker-based CTs. To our knowledge, this is the first study addressing this gap in evidence.

## Materials and Methods

### Study Design

This is a descriptive, prospective and longitudinal single-center study.

### Study Site and Population

The study will be conducted at the Clinical Emergency Hospital of Bucharest, Romania, a tertiary center which provides state of the art care to patients with various acute or chronic cardiac pathologies. A total of 333 patients admitted to the Cardiology Department will be enrolled starting with July 1, 2018. Patients ≥ 18 years old, of both genders and different demographic, social and educational backgrounds are eligible. The sample size to be included in the study was calculated using Power and Precision Software for the population of patients hospitalized in our department, which is around 4400 annually. In order to estimate the percentage of patients who would respond affirmatively to clinical study enrollment, which represents the main focus of our analysis, the sample size computation was based on the following assumptions: 1) the expected pattern of responses to clinical study enrollment based on our previous experience was considered as follows: 70% affirmative response, 25% negative response and 5% undecided; 2) we assumed that the percentage of missing data would be 3% based on our previous experience; 3) we took into consideration a confidence level of 95% and an error margin of plus/minus 5 points. Participants will be recruited until the proper sample size will be achieved. Individuals from vulnerable categories are of special interest, keeping in mind the importance of assuring equity in terms of access to medical research and care.

The study was approved by the local research ethics board. Of note, all participants will be interviewed only after signing the written informed consent; subjects’ autonomous decision will be respected, and their anonymity will be protected according to current EU legislation. Detailed explanations in plain language and some well-known examples of biomarkers have been offered in the ICF to be signed before completing the questionnaire.

### Questionnaire Development

A semi-structured, interviewer-administered questionnaire was created based on literature review [4, 7]. The interviewer-administered approach has been chosen due to the advantages it presents in communication with patients, such as fewer missing data and the opportunity to clarify any misunderstandings in questions or response options [8]. A panel of health professionals, comprising 2 senior cardiologists and 1 senior clinical psychologist refined the questionnaire accordingly.

The study questionnaire (Appendix A) encompasses the following sections: demographics (7 items), personal medical history (5 items), attitudes (9 items) and trust (4 items). Sociodemographic data to be recorded refers to respondents’ age, gender, place of residence and education. Ethnicity, religion and marital status will also be asked. Medical history will cover the existence of hospitalizations in the last 12 months, the presence of a chronic illness, the compliance to prescribed treatment and prior participation in medical studies. The impact of the illness on quality of life will be graded on a 10-point scale. The questionnaire also explores the motivations which might lie behind the individual choice to participate in biomarker-based clinical trials. Possible answers include – but are not limited to – personal benefit, and altruistic reasons – either to help other un/related people who might have the same disease in the future or to contribute to research advancements. Another category of questions refers to the extent of information to be disclosed, such as the purpose of the study explained at length, statistics regarding the importance of biomarkers, and drawings/schemes of relevant data. Trust in the doctors involved in research is an important part of the decision-making process; hence respondents will be asked to rate their opinions on a 5-point scale regarding participant safety, fidelity to the proper purpose of the study, and honesty about the conducted research.

### Data Collection

Data will be collected by trained medical staff. Every participant will be assigned a unique study research identification code. No protected personally-identifiable information will be part of the electronic database.

### Statistical Analysis

Data will be expressed as percentages for categorical variables and as the mean ± standard deviation for numeric variables. Descriptive statistics will be used to illustrate patients’ demographics, medical history, attitudes toward biomarker-based CTs and trust in medical researchers. Logistic regression models will be employed to assess relations between patients’ attitudes and trust and different socio-demographic variables. No substitutions will be made for missing data and analyses will be based on existing data only. Statistical analysis will be performed using SPSS software version 23.

## Discussion

Achieving the targeted number of participants is one of the main challenges faced when conducting a CT. An empirical analysis published in 2015 reported that almost one-fifth of 2579 trials were either terminated for failed accrual or completed with less than 85% expected enrolment [9]. Moreover, 86% of CTs do not complete recruitment targets within the specified time interval. Without sufficient participation, the number of subjects may prove to be too small to obtain accurate, conclusive results. As a result, constant efforts have been made to identify barriers to patients’ recruitment and retention, and appropriate strategies to overcome them have been addressed [10, 11]. Recently, the Clinical Trials Transformation Initiative Recruitment Project Team examined the challenges related to trial recruitment and issued actionable, evidence-based recommendations aiming to improve enrollment irrespective of disease or intervention [12]. One of the recommendations is to develop and test tailored messages on key points related to the study. This approach has already been employed by researchers aiming to design ICF based on patients’ feedback [1-5, 13]. It is common knowledge that IC is mandatory for a subject’s participation in research [14]. The role of ICF is to disclose information related to key aspects of the study, in order to enable a proper and knowledgeable decision. Basic data to be provided comprises the goal of the study, the procedures to be undergone and the potential risks and discomforts. To ensure freely given consent, statements indicating that participation is entirely voluntary and that refusal to accept will not involve any penalty or any loss of benefits should be made. Furthermore, the individual’s right to confidentiality and the right to withdraw or opt-out of the study at any time without any consequences should also be stated.

Knowing the rationale for people’s willingness to participate or not in CTs is imperative, but reasons can vary depending on the individual’s background. Literature research has revealed altruistic and/or hopeful outlook as main motives of participation [7]. Consequently, the purpose of the study should be clearly stated, as well as the participant’s benefits – if any. Conducted surveys exposed that awareness and consent expectations vary by the socio-demographic characteristics of the study population [15-17]. Therefore, the amount of information to be disclosed, as well as its presentation, may be tuned according to patients’ specific needs.

Adjusting ICF content is particularly important when specific subpopulations are targeted. For example, it has been shown that many racial and ethnic minority participants have an insufficient understanding of the information provided in relation to CTs in which they are involved [16, 18]. Partaking of individuals from vulnerable categories is, thus, critical in order to improve equity in access to medical research and care. Lack of participation may lead to a lack of sufficient information which will eventually be translated into suboptimal quality of care in terms of disease prevention, early detection, and treatment. The emphasis and extent of the information disclosed may need to be tuned to achieve equal representation in biomedical research.

### Questions Raising Ethical Implications

**Figure 1:**
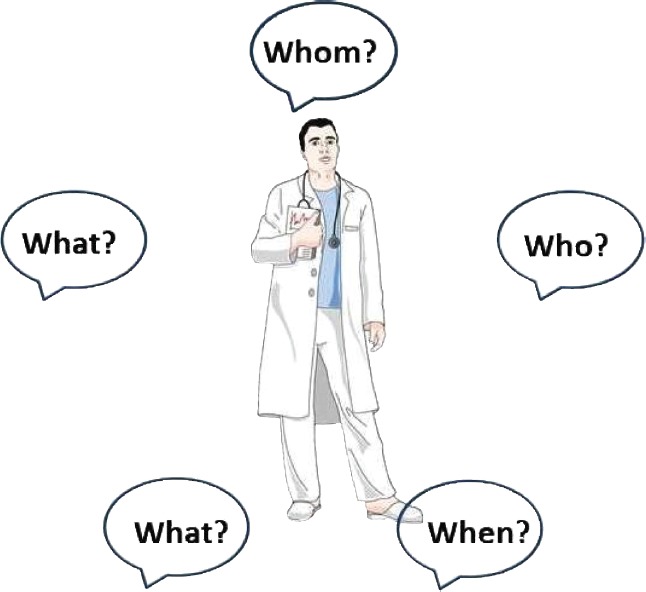
The 5 “Ws” raising ethical implications. This figure was produced using Servier Medical Art, available from www.servier.com/.

While biomarker-based CTs are acknowledged as valuable tools for a better understanding of the mechanisms underlying a patient’s condition, they also have major ethical implications. We identified 5 questions raised in order to balance an adequate message in inviting and gaining voluntary informed consent (Figure 1).

**Whom** should we ask to participate in biomarker-based CTs in order to ensure socio-demographic diversity?

We consider efforts should be made to include subjects from every social category and local minorities.

**Who** should make the invitation?

Indeed, one should keep in mind the power imbalance between patients and their treating doctor. We consider every effort should be made to avoid an excessive perception of pressure while being asked for consent. So, we addressed this particular issue by designing a query which explores the dynamics of the relationship between trust, doctor’s authority and pressure perception.

**When** should we invite the patients to participate? There is no simple answer to this question as the one-size-fits-all scenario does not apply. On the one hand, in the acute phase of a cardiovascular condition, patients are often under extreme stress and require urgent therapeutic interventions. On the other hand, the exact acute settings need to be further characterized. In this view, we consider that, excluding the circumstances when biomarkers related to the supra-acute/acute phase of a condition are studied, it is appropriate to delay asking for consent until the patient is stabilized.

**What** amount of information and in **what** form should it be disclosed in order to offer “sufficient information and adequate understanding of both the proposed research and the implications of participation in it”?

The patients will be kindly required to choose 6 out of 23 assertions which reflect their opinions regarding specific information.

### Addressing Gaps in Evidence

Survey data analysis will empower an in-depth understanding of patients’ perception which will ultimately lead to a tailored approach to patients’ enrollment in future biomarker-based CTs in our clinic.

## Conclusion

Herein we presented the methodology of a study designed to explore the attitude of patients from a tertiary cardiology center towards participation in biomarker-based clinical trials. We aim to approach the patients ethically and to increase the participation rate in future studies conducted in our clinic. To the best of our knowledge, this is the first study addressing this issue in Romanian patients. The information gained will contribute to developing tailored informed consent forms which will optimally suit local patients with different demographic, social and educational backgrounds.

### Sources of Funding

This work was supported by a grant of the Romanian National Authority for Scientific Research and Innovation, CNCS/CCCDI UEFISCDI, project number PN-III-P2-2.1-PED-2016-1333, within PNCDI III.

## Conflict of Interest

The authors confirm that there are no conflicts of interest.

## Questionnaire on the attitude of patients admitted in Clinical Emergency Hospital of Bucharest towards the participation in biomarker-based clinical trials

1. Identification code (*to be completed by the investigator in order of recruitment)*: Q-
2. Age:
3. Sex:
  ◻ M
  ◻ F
4. Residence:
  ◻ Urban
  ◻ Rural
5. Education:
  ◻ Higher education
  ◻ Secondary education
  ◻ High school
  ◻ Primary school/Gymnasium
  ◻ No degree
6. Ethnicity:
  ◻ ………………….
  ◻ I don’t know/I don’t answer
7. Religion/ Faith:
  ◻ ………………….
  ◻ I don’t know/I don’t answer
8. Marital status:
  ◻ Married
  ◻ Single
  ◻ Divorced/separated
  ◻ Widow
  ◻ I don’t answer
9. Any condition for which you have been hospitalized in the last 12 months?
  ◻ Yes, cardiovascular disease
  ◻ Yes, another condition……………………………………………………
  ◻ No
10. Are you diagnosed with a chronic illness?
  ◻ Yes, cardiovascular disease
  ◻ Yes, cancer
  ◻ Yes, another condition..…………………………………………………..
  ◻ No
11. Are you taking medication as prescribed?
  ◻ Yes
  ◻ No
  ◻ It is not the case
12. Have you ever participated in a medical study?
  ◻ Yes
  ◻ No
13. Impact of the illness on quality of life – on a scale of 1 to 10 (1 being deeply altered quality of life and 10 being excellent quality of life). How would you grade your quality of life at the present moment?**
  1  2  3  4  5  6  7  8  9  10
  ***by quality of life we define the perceived physical, mental and social well-being*
14. Do you consider valuable a clinical study using biomarkers?
  ◻ Yes
  ◻ No
  ◻ Impartial
    In the next section we refer to the hypothetical situation that you will be asked to participate in a biomarker-based clinical trial (for example: blood products, tissue or blood cells, genes/DNA).
15. Are you interested in participating in this study?
  ◻ Yes
  ◻ No
  ◻ Impartial
16. What is the main reason for accepting to participate in this study?
  ◻ Helping other patients who have the same disease as I have
  ◻ Helping my family members that could have the same disease as I have in the future
  ◻ Maybe the resulting information will be helpful for me
  ◻ In order to contribute to acquisition of knowledge about my disease, even if my family and I would not have any direct benefits
  ◻ In order to contribute to research
  ◻ Another reason:………………………………………………………………
  ◻ I would not participate
17. What information do you consider important in order to convince you to participate in this study? *Check two options*
  ◻ The possibility of finding out the study results and their impact on my health
  ◻ A non-invasive sample collection for the study
  ◻ Financial compensation for participants
  ◻ Knowing how the samples collected will be used
  ◻ Knowing what are the benefits of participating in the study
  ◻ The certainty of improving my well-being in the future as a result of participating in the study
  ◻ Ensuring that my samples will not be used in other ways than those mentioned
  ◻ Ensuring that my rights will be respected during the whole study
  ◻ The possibility of withdrawal from the study without affecting the quality of medical services
  ◻ Other:
  ◻ Nothing
18. If you would participate in this study, which of the following items do you find important for you? *Check two options*
  ◻ To be clearly stated the potential risks that I am exposed to
  ◻ The purpose of the study explained at length, in easy terms
  ◻ Drawings, schemes, tables with relevant data
  ◻ Statistics regarding the importance of biomarkers
  ◻ The possibility to communicate directly with the research team
  ◻ Other:………………………………………………………………………....
  ◻ Nothing
19. Which of the following items do you consider not useful to be provided when your participation agreement is requested? *Check two options*
  ◻ Long and complicated sentences /the lack of explanations in easy terms
  ◻ Insufficient medical information
  ◻ Too much medical information
  ◻ Insufficient information regarding your rights (the possibility of withdrawal from the study without affecting the quality of the medical services)
  ◻ Other:………………………………………………………………………….
20. Your trust or your participation in the study would be increased if the participation agreement would be asked by:
  ◻ Your treating doctor (the doctor who is treating your current condition)
  ◻ The clinical study coordinator
  ◻ Both the treating doctor and the clinical study coordinator
  ◻ Any doctor involved in the study
  ◻ Under no circumstances
21. Do you consider useful this questionnaire? How would you grade it on a scale of 1 to 5 (1 being not useful at all and 5 being very useful).
  1  2  3  4  5
22. Under which of the following circumstances would you agree to be contacted?
  ◻ For completing the information from this questionnaire
  ◻ For other studies conducted at Clinical Emergency Hospital of Bucharest, Cardiology Departament
  ◻ Under no circumstances
    Please tell us what is your opinion regarding the following statements:
23. The doctors involved in research wish only the well-being of each patient.
  ◻ I completely disagree
  ◻ I disagree
  ◻ Neutral/indifferent
  ◻ I agree
  ◻ I completely agree
24. The doctors provide the patients all the information/all it is necessary to know about the study.
  ◻ I completely disagree
  ◻ I disagree
  ◻ Neutral/indifferent
  ◻ I agree
  ◻ I completely agree
25. I completely trust the doctors who are involved in medical research
  ◻ I completely disagree
  ◻ I disagree
  ◻ Neutral/indifferent
  ◻ I agree
  ◻ I completely agree
26. The doctors involved in research treat the patients as “objects”.
  ◻ I completely agree
  ◻ I agree
  ◻ Neutral/indifferent
  ◻ I disagree
  ◻ I completely disagree
27. Is there anything else you would like to add for this questionnaire (comments, sugestions)?
  …………………………………………………………………………………………
  …………………………………………………………………………………………

Thank you for the participation,

QUEST team.
